# Managing Innovation for Sustainable Health:rethinking training of health officials in fragile states: Freddy Bangelesa

**DOI:** 10.1093/eurpub/ckac131.101

**Published:** 2022-10-25

**Authors:** K Båge, C Batte, L Tomasdotter, M Mala Ali, M Alasow, R Ndejjo, D Hellden, G Ndeezi, T Alfven, R Wanyenze

**Affiliations:** 1 Global Public Health, Karolinska Institutet, Stockholm, Sweden; 2 School of Public Health, Makerere University, Kampala, Uganda; 3 Head of Sweden Office, Tinkr, Stockholm, Sweden; 4 Kinshasa School of Public Health, University of Kinshasa, Kinshasa, Democratic Republic of the Congo; 5 Faculty of Health Science, Benadir University, Mogadishu, Somalia

## Abstract

There is great urgency for action to achieve the Sustainable Development Goals, especially in fragile settings, which face acute and complex challenges. Yet, the public sector may be limited in its capacity to address these appropriately, with devastating effects on the health of people and environment now and in the future. The challenges to sustainable health require professionals who are trained relevant competences. In 2020, Karolinska Institutet, Sweden, and Makerere University, Uganda, developed the Centre of Excellence for Sustainable Health under which a new partnership was established with Benadir University, Somalia, Kinshasa School of Public Health, the Democratic Republic of Congo, and Tinkr, Norway to develop training on innovation for sustainable health. The aim of “Managing Innovation for Sustainable Health” (MISH) is to strengthen the capacity to contribute to achieving sustainable health through innovation in the public sector. It targets managers in Somalia, DRC and Uganda from the public and private sector, academia, and civil society. It is one year long, part-time and delivered online with one study trip. It features three modules covering Agenda 2030 and Sustainable Health; Multisectoral Collaboration and Implementation Science; and Innovation and Innovation Management. Integration of participants’ learnings into their professional role, mutual learning between participants, and an emphasis on applicability, all underpin the learning strategies of the program. Quality is monitored through expectation surveys, baseline mid and final impact assessments, module and final program evaluations. MISH has trained about 50 managers, 85% of which say that the training was useful. All partners are represented in both operational and strategic organizational bodies of the program. Our model shows what is possible through collaborative online international learning delivered by partnerships defined by teamwork, trust, and a dedication to true impact.

**Key messages:**

• There is great urgency for action to achieve sustainable health especially in fragile settings.

• There is momentum for higher education to leverage the opportunities of the covid-19 to rethink learning for the global challenges.

